# Floral Volatiles in Parasitic Plants of the Orobanchaceae. Ecological and Taxonomic Implications

**DOI:** 10.3389/fpls.2016.00312

**Published:** 2016-03-15

**Authors:** Peter Tóth, Anna K. Undas, Francel Verstappen, Harro Bouwmeester

**Affiliations:** ^1^Laboratory of Plant Physiology, Wageningen University and Research CentreWageningen, Netherlands; ^2^Department of Plant Protection, Slovak University of Agriculture in NitraNitra, Slovakia; ^3^RIKILT, Wageningen University and Research CentreWageningen, Netherlands

**Keywords:** broomrapes, *Orobanche*, *Phelipanche*, volatile organic compounds, weeds, taxonomy, floral scents, phylogenetic patterns

## Abstract

The holoparasitic broomrapes, *Orobanche* spp. and *Phelipanche* spp. (Orobanchaceae), are root parasites that completely depend on a host plant for survival and reproduction. There is considerable controversy on the taxonomy of this biologically and agronomically important family. Flowers of over 25 parasitic Orobanchaceae and a number of close, parasitic and non-parasitic, relatives emitted a complex blend of volatile organic compounds (VOCs), consisting of over 130 VOCs per species. Floral VOC blend-based phylogeny supported the known taxonomy in internal taxonomic grouping of genus and eliminated the uncertainty in some taxonomical groups. Moreover, phylogenetic analysis suggested separation of the broomrapes into two main groups parasitizing annual and perennial hosts, and for the annual hosts, into weedy and non-weedy broomrapes. We conclude that floral VOCs are a significant tool in species identification and possibly even in defining new species and can help to improve controversial taxonomy in the Orobanchaceae.

## Introduction

Just as humans and other animals use sound to communicate with organisms in their environment, flowering plants have a sophisticated language for communication, floral scents or floral volatile organic compounds (VOCs). Flowers often emit hundreds of different VOCs and there are large differences between species (Pichersky and Gershenzon, 2002; Raguso, 2008a). These complex blends present a detailed language for communication with other organisms (Dudareva et al., 2006). Floral scent is one of the adaptations that plants have evolved to attract and guide pollinators (Kaiser, 2006; Raguso, 2008b), but VOCs act as a filter at the same time (Junker et al., 2010). The scent of flowers of different species is never the same. This is caused by the large diversity of VOCs and their relative abundance (Knudsen et al., 2004). So far, over 1700 compounds have been identified in floral blends (Knudsen et al., 2006) and one species may emit from one to over a hundred different compounds (Knudsen and Gershenzon, 2006). This implies that the flower VOC blend contains an enormous amount of information (Dudareva and Pichersky, 2006), potentially also on taxonomic relationships. However, only a few studies report on their significance in phylogeny. In male euglossine bee-pollinated orchids, floral fragrance data did not reliably indicate phylogeny (Williams and Whitten, 1999). In two genera of Nyctaginaceae, and in four *Licuala* species (Arecaceae) flower VOCs did not reflect classification (Levin et al., 2003; Meekijjaroenroj et al., 2007). While phylogenetic relations based on major floral VOCs revealed no distinct patterns at higher taxonomic levels (Knudsen et al., 2006), phylogenetic patterns were detected in *Cypripedium* (Orchideaceae) (Barkman, 2001) and within *Nicotiana* (Raguso et al., 2006). In the present study we explored the possibilities of using flower VOCs as phylogenetic markers in the taxonomically complicated group of parasitic flowering plants, the broomrapes (Orobanchaceae). These root parasitic plants have significant impact on the ecosystem and often severely reduce host performance (Press and Phoenix, 2005). It has been suggested that the host range and life history of the broomrapes evolved in a concerted fashion (Schneeweiss, 2007) and that the ancestor broomrape had a narrow host range (Manen et al., 2004). However, species with a wide host range have evolved (Parker, 2009), and mostly grow as weeds on annual crops (Rubiales et al., 2009), while the broomrapes in natural ecosystems usually have only one or a few, usually perennial, hosts (Teryokhin, 1997).

The latest monograph of the *Orobanche sensu lato* (Beck-Mannagetta, 1930) and current keys (Teryokhin et al., 1993) stress floral characters as species-discriminative characteristics. These morphological traits are difficult to determine (Musselman et al., 1981). Host specificity is therefore often employed as a major factor in classification. To better establish taxonomic relationships various other approaches have been employed. Nuclear rDNA internal transcribed spacer (ITS) sequence data (Wolfe et al., 2005), DNA sequences of *rbc*L (large subunit of the enzyme ribulose-1,5-bisphosphate carboxylase), *ndh*F (NADH dehydrogenase subunit 5), the plastidic *rps* (ribosomal proteins) (Tank et al., 2006), *rps2* (ribosomal proteins 2) (Park et al., 2008), phytochrome A (*PHYA*) (Bennett and Mathews, 2006) and the low-copy nuclear locus (PHYB) (McNeal et al., 2013) have been used to discriminate genera. Random amplified polymorphic DNA (RAPD) analysis (Román et al., 2003), proteomics (Castillejo et al., 2009), amplified fragment length polymorphism (AFLP) analysis (Vaz Patto et al., 2009), more detailed plastid *rbc*L sequencing (Manen et al., 2004) and nuclear ITS sequences (Schneeweiss et al., 2004; Carlón et al., 2005, 2008) were employed to discriminate species. Only the last three studies dealt with the Old World *Orobanche* genus as a whole. It was split into an *Orobanche* and a *Phelipanche* genus. Nevertheless the phylogenetic relationships remain still insufficiently understood and controversial (Park et al., 2008).

The present study offers insight into the floral scent chemistry of Orobanchaceae. We used ultra-sensitive, unbiased, floral headspace metabolite profiling and advanced data-analysis and statistical methods normally applied in metabolomics. The floral volatile blends of 32 species from the root parasitic plant genera *Orobanche, Phelipanche, Boulardia, Cistanche, Striga*, and parasitic outgroup *Cynomorium* as well as the non-parasitic but related species from the genera *Antirhinum, Mimulus*, and *Paulownia* were analyzed and the VOC data used to infer taxonomic relationships. The results are compared with previously established phylogenetic relationships as based on taxonomic considerations or molecular markers.

We show that the analysis of the floral chemistry is a reliable to infer phylogenetic relationships within the Orobanchaceae as we can confirm relationships established based on other approaches. Moreover, our study highlights a number of disputed and/or unreliable phylogenetic relationships. We argue that floral scent can be used to distinguish species, which are difficult to discern based on morphological traits. Finally, our analysis suggests separation of the broomrapes into two different groups, of species growing on annual and short lived perennial hosts and species growing on perennial hosts, suggesting the correlation between VOC profiles and host features.

## Materials and Methods

### Plant Material

A total of 32 plant taxa were screened for floral VOCs. We focused on the two most important genera of Orobanchaceae, *Orobanche* (16 species) and *Phelipanche* (4 species). Other parasitic broomrapes from genera *Cistanche* (2 species), and *Boulardia* (1 species), and non-broomrape (but Orobanchaceae) parasitic species, *Striga* spp. (3 species) were included as well. In addition, six outgroup species, one non-Orobanchaceae parasitic *Cynomorium* sp. (1 species) and five closely related non-parasitic species, *Antirrhinum* spp. (2 species), *Mimulus* spp. (2 species), and *Paulownia* sp. (1 species) were used (see Supplementary Table S1). Of all species more than five samples were used for headspace collection as some samples, especially in the field, were influenced by water or had poor signal intensity. As it is difficult or sometimes impossible to cultivate some of the broomrapes under greenhouse conditions, VOCs of 18 broomrapes were trapped under natural conditions *in situ*. Four of them, iii, *O. caryophyllacea, O. flava*, and *O. reticulata* were checked in different locations and *O. lutea* in two different years. These are referred to within figures and tables using numbers, e.g., *O. alba* 1, *O. alba* 2 (Supplementary Table S1). The remaining 14 species were grown under greenhouse conditions as described by Kroschel (2001). The seeds were sown in a soil-sand mixture 3:1 in a top layer of approximately 3–10 cm deep. The seed density was 10 mg/L of soil. To break dormancy the pots with the broomrape seeds were watered daily with 60 ml of water, during 12 days at 16 h light/8h dark, 21/18°C, and 60% relative humidity (Matúšová et al., 2004). Subsequently, germinated host plant seeds were introduced to the pots and watered daily for 5 days, after which the watering regime was changed to three times per week until broomrapes flowered. The same conditions were used for non-parasitic plants. For more details about the species used in this study see Supplementary Table S1.

### Headspace/VOC Trapping

The VOCs emitted by flowers were collected using dynamic headspace sampling as described by Kappers et al. (2010). Two or three flowering shoots (=plants) or only flowers (in the case of non-parasitic plants) were cut, placed into vials filled with water and immediately transferred to a glass jar (720 ml), which was tightly closed with a Teflon lid with an in- and outlet. Air was drawn from the jar through a stainless steel cartridge (Markes, Llantrisant, UK) containing 200 mg Tenax TA (20/35 mesh; Grace-Alltech, Breda, The Netherlands) and the incoming air was purified by passing through a similar cartridge containing Tenax. In the field the air was drawn off the jar using the portable PAS-500 Micro Air Sampler (Supelco, Sigma–Aldrich) with Low Flow Orifice and in the greenhouse using electric pumps (ADC, The Analytical Development Co Ltd, Hoddesdon, England). The air flow was controlled by a flow controller (Brooks Instr. Veenendaal, The Netherlands) which was set to approximately 100 ml min^-1^. VOCs were trapped for 5 h, mostly in the morning from 9:30. Four to six collections and its respective control (empty glass jar) were made simultaneously. After sampling, the Tenax cartridges were capped and stored under room temperature until analysis.

### Gas Chromatography – Mass Spectrometry (GC–MS) Headspace Analysis

Analysis of the trapped volatiles was performed as described by Snoeren et al. (2010) with some modifications on a Trace Ultra GC coupled to a Thermo Trace DSQ quadrupole Mass Spectrometer (Interscience, The Netherlands). Control samples (blank Tenax cartridges) were used to disqualify any non-biological compounds during each GC run. Trapped VOCs were released from the Tenax by heating the cartridge at 250°C for 4 min with a helium flow of 30 ml min^-1^ and re-trapped on a multi-bed sorbent at 10°C. By heating this sorbent, the compounds were injected into a capillary column (RTX-5MS, 30 m, 0.25 μm id, 1.4 μm df, Restek, Interscience, The Netherlands). The GC temperature program started at 60°C (hold for 2.5 min) and rose to 250°C at 12°C min^-1^ (hold for 1.5 min) with a column flow of 1 ml min^-1^. The column eﬄuent was ionized by electron impact ionization at 70 eV. Mass spectra were collected over the mass range *m/z* 35 to 300 at a scan rate of three spectra sec^-1^. The chromatography and spectral data were evaluated using Xcalibur software (v. 2.0.7, Thermo Fisher Scientific Inc.). Compounds were annotated by comparing the mass spectra with mass spectral libraries (Wiley 7th edition and NIST08), and by comparing calculated retention indices with those given by NIST08, Adams (2007) and El-Sayed (2012). The annotation of many compounds was verified using an in house developed mass spectra/RI library. Any suspicious compounds (e.g., benzene, toluene, 1,4-dichlorobenzene) were carefully checked in the literature and in The Pherobase (El-Sayed, 2012) for known biological properties and occurrence in other plants, to be accepted as flower VOCs or at least potential volatiles as there are no data about in connection with broomrapes in the literature.

### Data Analysis

Prior to statistical analysis the entire set of chromatograms was baseline corrected and aligned using Metalign software version 010110 (Lommen, 2009). Masses that were present in less than 5 samples and had a signal below 100000 were removed using an in-house script called MetAlign Output Transformer (METOT; Plant Research International, Wageningen, The Netherlands) and mass spectra reconstituted using MsClust (Tikunov et al., 2005). In this way Metalign output was reduced from 16366 mass peaks to 639 putative compounds. Non-biological chemicals were traced using Xcalibur software (v. 2.0.7, Thermo Fisher Scientific Inc.) and removed from the dataset. Samples that were too much influenced by polysiloxanes (column related compounds with m/z 207, 267, 355, etc.) or water, or had poor quality (signal intensity) were excluded from the dataset. Data, peak intensities obtained after Metalign were^2^ log transformed and the mass signals normalized by subtraction of the average peak height of all signals of that mass across all samples from each individual signal for that mass (mean centering). Principal component analysis (PCA) was performed on compounds whose peak heights were significantly different between taxa *(P* < 0.05 as determined using ANOVA) using GeneMath XT (v. 2.12, Applied Maths NV, Belgium). Hierarchical clustering analysis (HCA) with 10000 bootstrap re-samplings was performed in R (v. 2.10.1; R Development Core Team) following Shimodaira (2004).

## Results

### Floral Scent Chemistry

**FIGURE 1:**
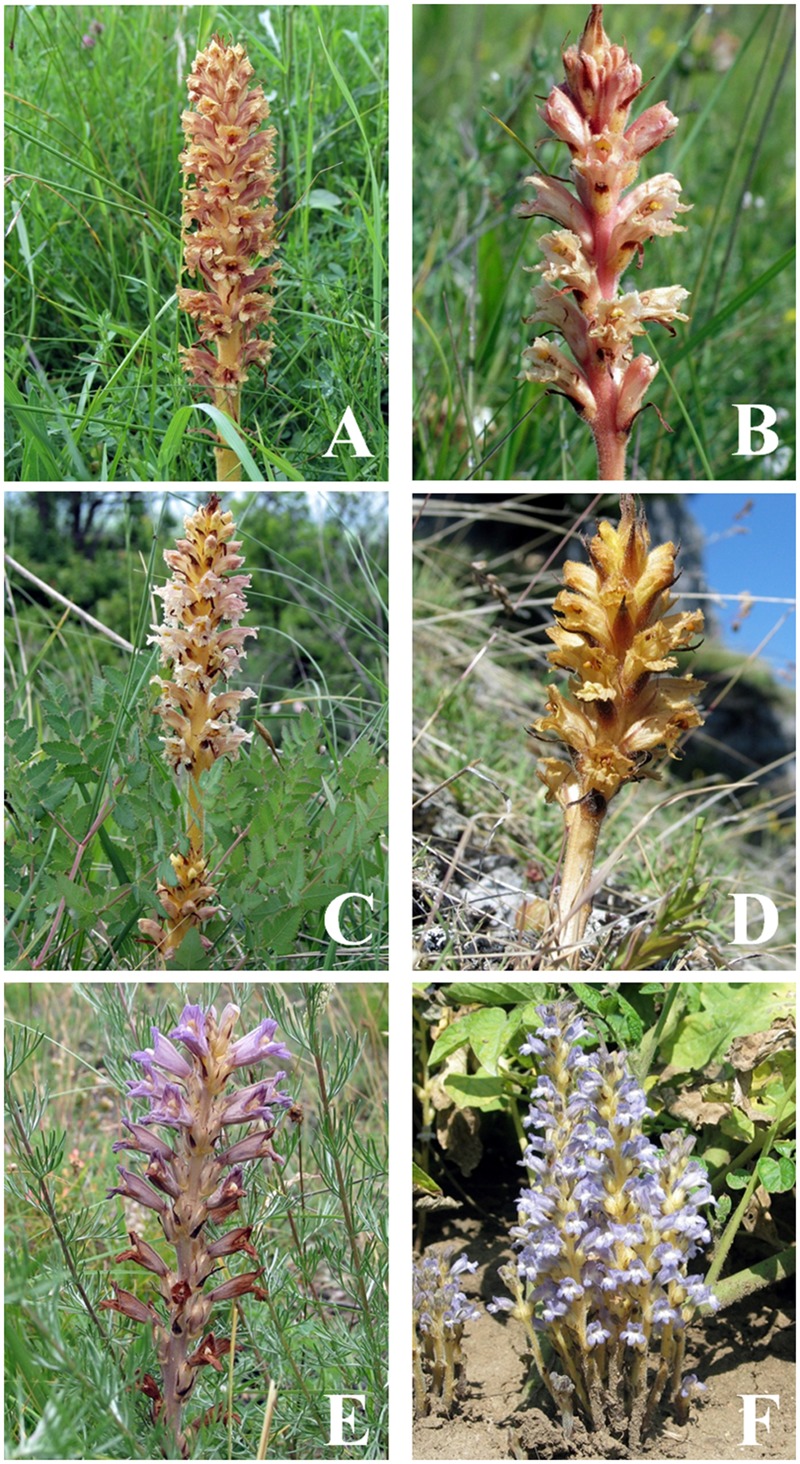
**Examples of some morphologically, taxonomically, and evolutionary difficult to distinguish broomrape species, **(A)***Orobanche elatior*, **(B)***Orobanche kochii*, **(C)***Orobanche alsatica*, **(D)***Orobanche mayeri*, **(E)***Phelipanche arenaria*, and **(F)***Phelipanche ramosa***.

Dynamic headspace sampling was performed either in the greenhouse or headspace collection chamber (14 plant species) or in the field (18 species). All the headspace samples (at least five per species) of the 32 plant species (Supplementary Table S1) were analyzed using GC–MS. The entire GC–MS dataset allowed the detection of 639 compounds of which 482 were significantly different between any of the species analyzed. Most of these compounds could be (putatively) identified. These data are completely novel as this is the first report of VOCs for any holoparasitic Orobanchaceae. The significantly different metabolites were used for further analysis using unsupervised PCA. For four *Orobanch*e spp. (*O. alba, O. elatior*/**Figure 1A**/, *O. flava*, and *O. reticulata*), two *Phelipanche* spp. (*Ph. aegyptiaca* and *Ph. ramosa*/**Figure 1F**/) and two outgroup species (*Mimulus luteus* and *Paulownia tomentosa*) a detailed annotation of the floral VOCs is shown in Supplementary Table S2. In these six broomrapes, 393 VOCs could be tentatively identified, and although each species was characterized by a different VOC composition, there was also considerable overlap (Supplementary Table S2). A total of 59 from 278 metabolites were present in the blend of all four *Orobanche* spp., 95 from 219 compounds were present in both *Phelipanche* spp. and 43 VOCs were common in all six broomrapes shown in Supplementary Table S2. The *Phelipanche* spp. emitted about 13% more VOCs than the *Orobanche* spp. Floral scents of outgroup species represented rather simple blends in comparison with broomrapes, containing only from 66 (*M. luteus*) to 71 compounds (*P. tomentosa*) (Supplementary Table S2). Spectrum of VOCs across all 20 holoparasitic broomrape species (*Orobanche* spp. and *Phelipanche* spp.) was very rich and unusually diverse, including representatives of at least 12 functional groups: alcohols, aldehydes, amines, hydrocarbons (including monoterpenoids and sesquiterpenoids), aromatic hydrocarbons, carboxylic acids, esters, furans, ketones, phenols, sulfur compounds, and diverse functional groups. Many compounds are known as semiochemicals with behavioral function (e.g., kairomones, attractants, and pheromones). Each species has its own characteristic VOCs profile. However, 40 compounds were presented in the scent of all 20 broomrapes studied (Supplementary Table S3). These mutual compounds were strongly dominated by hydrocarbons, which represented 37.5% of them, followed by aldehydes (17.5%), alcohols (15%), aromatic hydrocarbons (10%), ketones (10%), esters (5%), carboxylic acids (2.5%), and diverse functional groups (2.5%). Certain compound, as acetone, toluene, pentanal, dibutyl phthalate, 4-methylpentan-2-one, and many others within broomrape VOCs are unusual, however, already known from plants. The striking exception within mutual compounds features pentane-2,4-dione and 2-decen-1-ol which are known from no other plants so far. Very characteristic chemicals for most of broomrapes (except, e.g., *Ph. aegyptiaca*) are also isopropyl tetradecanoate and isopropyl hexadecanoate. There are many other interesting compounds emitted by broomrapes. Scent of some broomrapes is aromatic, e. g., strong clove-like odor of *O. alba* brings on 4-ethylguaiacol and 4-vinylguaiacol, very strong fusel-like smell of *O. foetida* causes tetradecane and tetradecanal, and earthy, mushroom fragrance of *Ph. aegyptiaca* is result of benzyl tiglate emission.

### Floral Scent Relations and Clustering

**FIGURE 2:**
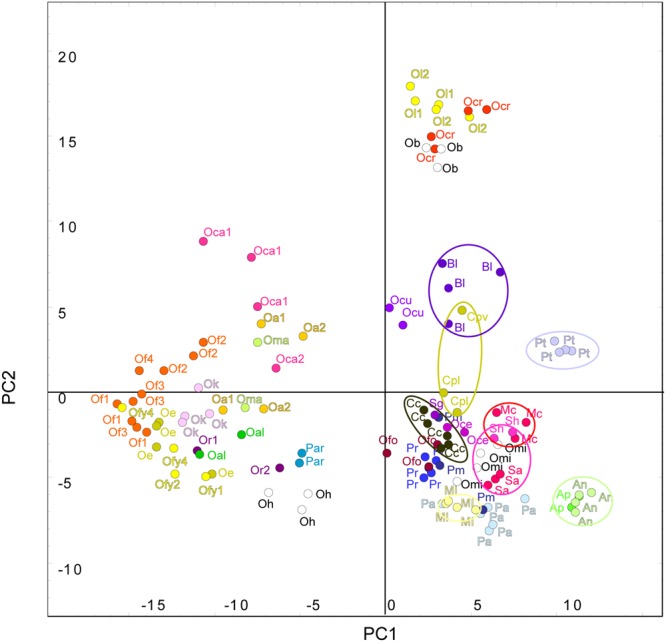
**Principal component (PC) analysis of the floral blends of the 32 species studied.** Principal component analysis (PCA) was done with compounds showing a significant difference between the species (*P* < 0.05 from ANOVA test) and it was performed by GeneMath XT. Letters and symbols represent single species. 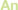, *Antirrhinum majus nanum*; 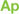, *A. majus pumilum*; 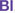, *Boulardia latisquama*; 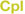, *Cistanche phelypea* (lutea); 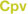, *Ci. phelypea* (violacea); **Cc**, *Cynomorium coccineum*; 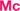, *Mimulus cardinalis*; 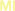, *M. luteus*; 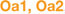 *Orobanche alba* (location 1, 2); 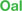, *O. alsatica*; **Ob**, *O. ballotae* (white color); 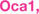 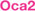, *O. caryophylacea* (location 1, 2); 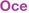, *O. cernua*; 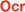, *O. crenata*; 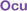, *O. cumana*; 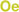, *O. elatior*; 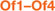, *O. flava* (location 1,2,3,4), 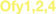, *O. flava* yellow morph (location 1,2,4); 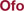, *O. foetida*; **Oh**, *O. hederae* (white color); 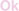, *O. kochii*; 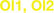, *lutea* (year 1,2); 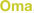, *O. mayeri*; **Omi**, *O. minor* (white color); 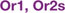, *O. reticulata* (location 1,2); 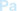, *Phelipanche aegyptiaca*; 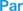, *Ph. arenaria*; 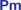, *Ph. mutelii*; 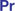, *Ph. ramosa*; 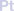, *Paulownia tomentosa*; 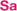, *Striga asiatica*; 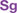, *S. gesnerioides*; 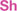, *S. hermonthica*.

In order to assess the total variation in the whole dataset of all species, the volatile profiles were analyzed by principal component analysis (PCA). PCA clearly showed the species differences in VOC composition, clustering the 32 species into separate groups, with the replicate samples of each species usually clustering close together (**Figure 2**). This is remarkable and demonstrating the reproducibility of our collection and analysis methods as some of the species were sampled in different locations and years. There was no or just negligible effect of trapping location on relative emission of VOCs. See for example *O. alba* 1–2, *O. flava* 1–4, *O. caryophylacea* 1–2, *O. reticulata* 1–2 (**Figure 2**). Also the year of sampling did not affect the volatile profile. See for example *O. lutea* 1–2 (**Figure 2**).

**FIGURE 3:**
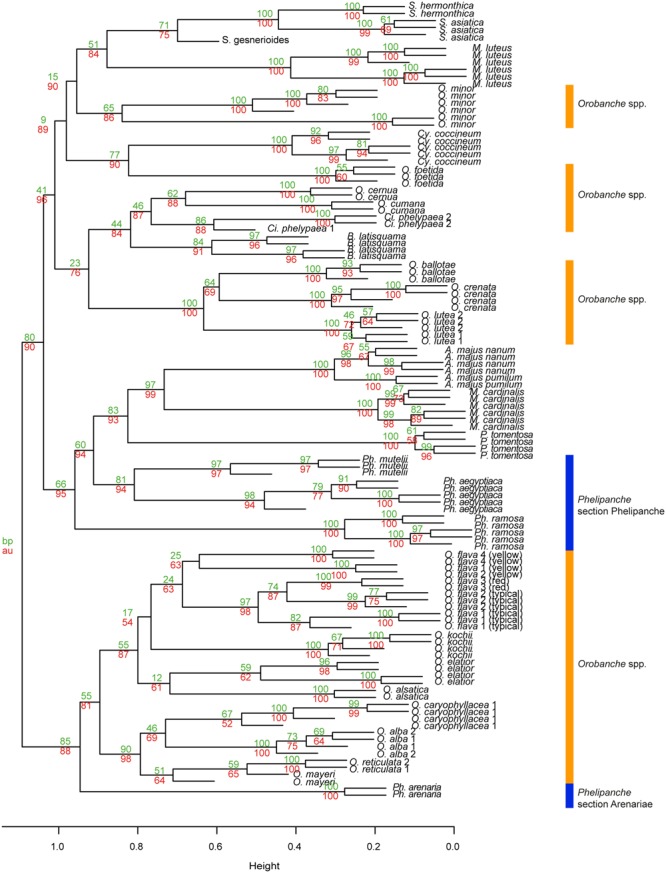
**Hierarchical clustering diagram of the flower VOC profiles of 32 species studied.** HCA was performed with R and based on PCA of significantly different flower blends. Numbers at nodes are bootstrap probability *P-*value (BP, upper green numbers) and approximately unbiased *P-*values (AU, lower red numbers). The definition of the two genera of *Orobanche* is designated in orange (genus *Orobanche*) and blue (genus *Phelipanche*) bar; section *Arenariae* is also indicated (subsections within genus *Orobanche* are shown in **Figure 6**).

**FIGURE 4:**
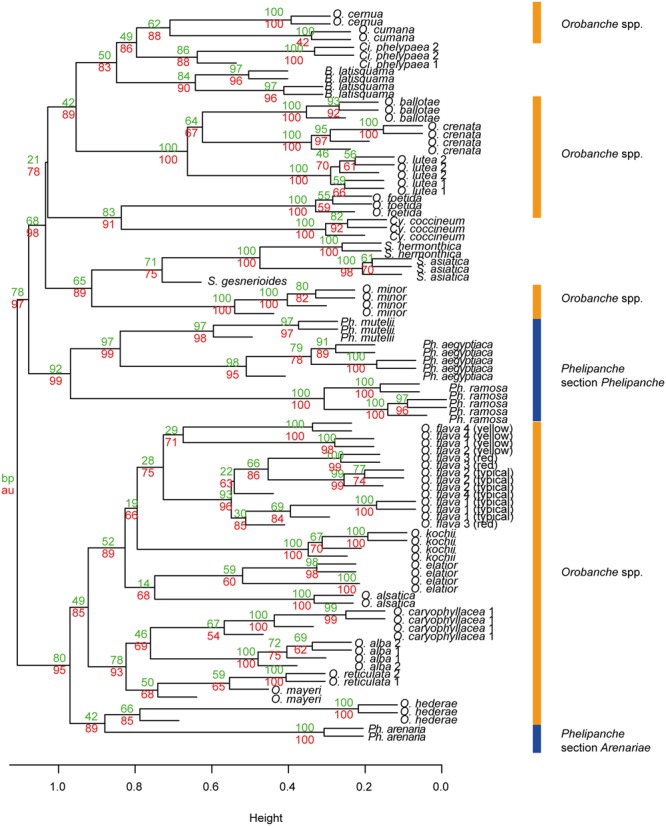
**Hierarchical clustering diagram of the flower VOC profiles of all parasitic plant species studied.** HCA was performed with R and it was based on PCA of significantly different flower blends after all non-parasitic plant species where removed from the data set. Numbers at nodes are bootstrap probability *P*-value (BP, upper green numbers) and approximately unbiased *P*-values (AU, lower red numbers). The definition of the two genera of *Orobanche* is designated by orange (genus *Orobanche*) and blue (genus *Phelipanche*) bars; section *Arenariae* is also indicated (subsections within genus *Orobanche* are shown in **Figure 6**).

The first three principal components (PCs) explained 25.6% of the observed variation in the VOCs while the first six PCs explained 39.6%. All analyses resulted in two distinct and highly supported groups. Broomrapes growing on perennial hosts (**Figure 2**, left side) clearly separated from the rest of the broomrapes growing on annual and short-lived perennial hosts as well as other parasitic plant species and non-parasitic plants (**Figure 2**, right side). The non-*Orobanche* parasitic *Boulardia latisquama*, and non-parasitic *P. tomentosa, Antirhinum majus nanum, A. majus pumilum*, and *M. cardinalis* clustered separately from each other and as group in between the parasitic annual host *Orobanche* (*O. cernua, O. cumana, O. foetida, O. minor*), *Phelipanche* (*Ph. aegyptiaca, Ph. mutelii, Ph. ramosa*) and *Striga* spp. (*S. asiatica, S. gesnerioides, S. hermonthica*). Non-parasitic *M. cardinalis* clustered closely with the non-broomrape parasitic *S. hermonthica*. Other non-broomrape parasitic species (*Cistanche phelypaea, Cynomorium coccineum*) cluster separately from most broomrapes but are quite similar (**Figure 2**). Hierarchical clustering analysis (HCA) showed that the *Phelipanche* species – with a remarkable exception for *Ph. arenaria* – share the same clade with the non-parasitic *A. majus nanum, A. majus pumilum, M. cardinalis*, and *P. tomentosa* (**Figure 3**). *O. cumana* and *O. cernua* cluster together with the non-broomrape parasitic species *B. latisquama* and *Ci. phelypaea*. *O. foetida* groups with the non-broomrape parasitic species *Cy. coccineum* and *O. minor* with the witchweeds (*Striga* spp.) and the non-parasitic *M. luteus* (**Figure 3**). The Approximately Unbiased *P*-value (AU, lower red numbers), obtained using Pvclust (a better approximation for reliability of clustering than the Bootstrap Probability *P*-value; BP, upper green numbers) shows that most clusters within the data set are reliable and significant (**Figure 3**). In order to explore how robust the clustering is, we analyzed the stability of the clustering after modifying the dataset. HCA on smaller subsets (e.g., without non-parasitic plants) resulted in the very similar output as the whole dataset (**Figure 4**). **Figure 4** shows that broomrapes growing on perennial hosts are further subdivided into a cluster containing the genus *Orobanche* and a cluster containing the genus *Phelipanche*, section *Arenariae* (bottom **Figure 4**). The same is true for the annual host broomrapes, where the genus *Phelipanche*, section *Phelipanche* is markedly separated from the genus *Orobanche* (top **Figure 4**). Based on the VOC data, *Orobanche* and *Phelipanche* are not monophyletic groups (taxon forming a clade).

### Phylogenetic Patterns within the Broomrapes

**FIGURE 5:**
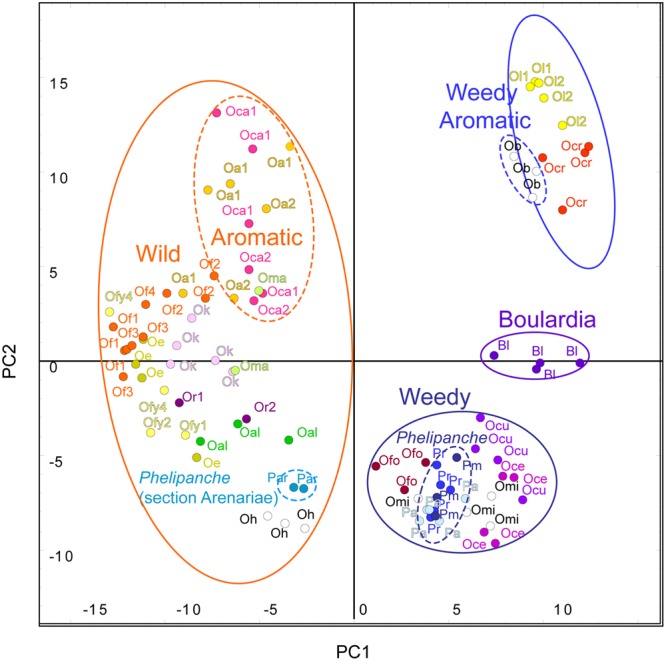
**Principal component analysis of the floral blends of the 21 European broomrapes studied (including *Boulardia latisquama*).** PCA was done with compounds showing a significant difference between the species (*P* < 0.05 from ANOVA test) and it was performed by GeneMath XT on a data subset with broomrapes only. Letters and symbols represent single species. Group of wild broomrapes: 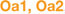 *Orobanche alba* (location 1, 2), 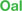, *O. alsatica*; 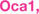 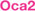, *O. caryophylacea* (location 1, 2); 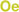, *O. elatior*; 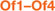, *O. flava* (location 1,2,3,4); 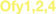, *O. flava* yellow morph (location 1,2,4); **Oh**, *O. hederae* (White color); 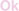, *O. kochii*; 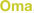, *O. mayeri*; 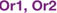, *O. reticulata* (location 1,2); 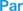, *Phelipanche arenaria*. Group of weedy broomrapes: **Ob**, *O. ballotae* (White color); 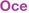, *O. cernua*; 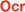, *O. crenata*; 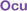, *O. cumana*; 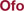, *O. foetida*; 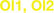, *O. lutea* (year 1,2); **Omi**, *O. minor* (White color); 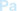, *Ph. aegyptiaca*; 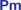, *Ph. mutelii*; 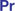, *Ph. ramosa*. Other broomrapes: 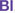, *Boulardia latisquama.*

**FIGURE 6:**
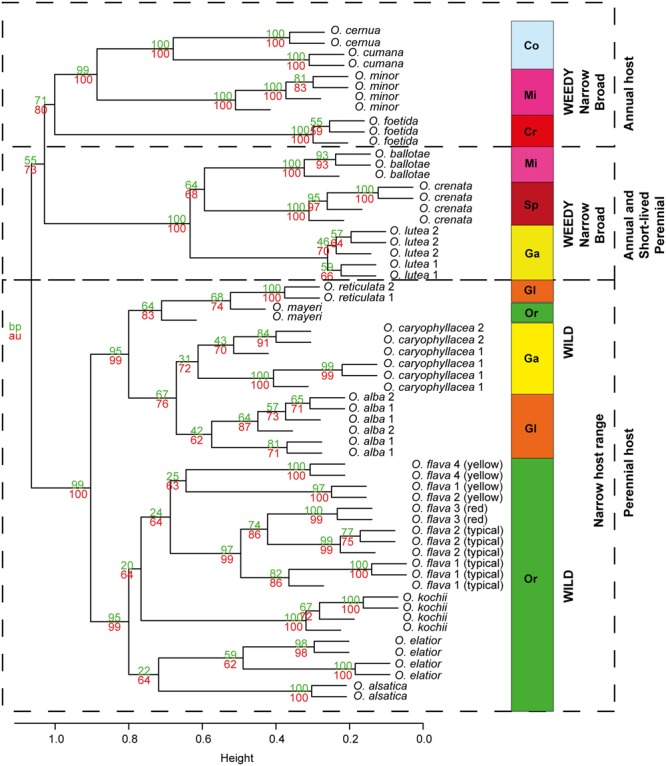
**Hierarchical clustering diagram of the flower VOC profiles of broomrapes of the genus *Orobanche*.** HCA was performed with R and it was based on PCA using a subset with data of *Orobanche* spp. only. Numbers at nodes are bootstrap probability *P*-value (BP, upper green numbers) and approximately unbiased *P*-values (AU, lower red numbers). Internal taxonomic grouping of genus *Orobanche* as proposed by Teryokhin (1997) is shown by letters and colors which indicate subsections (sections are not specified here), CO, *Coerulescentes*; CR, *Cruentae*; GA, *Galeatae*; GL, *Glandulosae;* MI, *Minores;* OR, *Orobanche*; SP, *Speciosae*.

**FIGURE 7:**
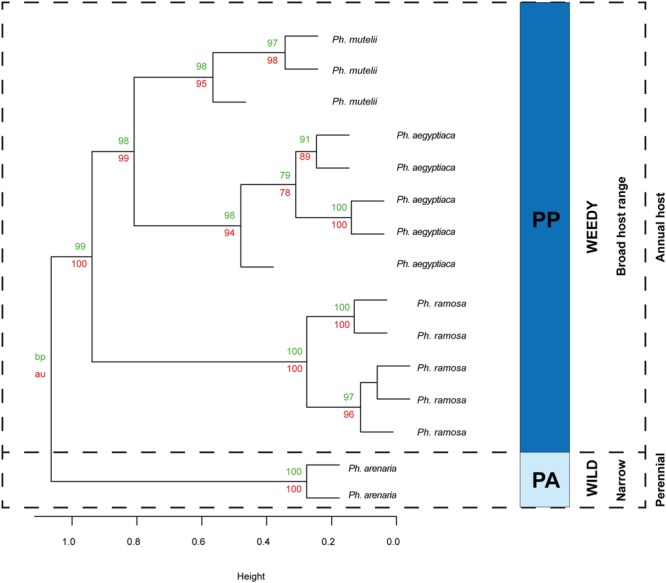
**Hierarchical clustering diagram of the flower VOC profiles of all broomrapes of the genus *Phelipanche*.** HCA was performed with R and it was based on recalculated PCA using a subset with data of *Phelipanche* spp. only. Numbers at nodes are bootstrap probability *P*-value (BP, upper green numbers) and approximately unbiased *P*-values (AU, lower red numbers). Taxonomic grouping of the genus *Phelipanche* as proposed by Teryokhin (1997) is shown by letters and colors which indicate sections, PP, *Phelipanche* and PA, *Arenariae.*

To visualize the phylogenetic relationships between the broomrapes in more detail, PCA was done on the VOC data of *Phelipanche* and *Orobanche* using *Boulardia* as an outgroup (**Figure 5**). The broomrapes were divided along the first PC (explaining 11.2% of the variation) into two groups, the perennial-host- and the annual-host-broomrapes. The first group comprises species exclusively growing in the wild; the second group comprises mostly weedy broomrapes. The statistically well supported position of *Ph. arenaria* (between *Orobanche* spp.) is particularly surprising. The annual-host broomrapes are subdivided into two groups along the second PC (explaining 19.8% of variation) into a cluster containing the aromatic (=strong fragrant VOC blend) weedy species *O. crenata, O. lutea*, and the non-weedy and aromatic *O. ballotae* and a cluster containing all other weedy species of the genera *Orobanche* (*O. cumana, O. cernua, O. minor, O. foetida*) and *Phelipanche* (*Ph. aegyptiaca, Ph. mutelii, Ph. ramosa*) (**Figure 5**). **Figures 6** and **7** illustrate the association of host specificity and host life history on phylogenetic relationships. The weedy cluster comprises almost exclusively species growing on annuals or short-lived perennial hosts. Most of these species are weeds in agriculture, except *O. ballotae* and *B. latisquama*. The latter is growing on a perennial host. *Boulardia* is not, but was in the past regarded to be an *Orobanche* spp., so we included it here as a closely related out-group. The host range of the weedy group ranges from monophagy (*O. ballotae, O. cumana, O. lutea*) through broad oligophagy (*O. cernua, O. foetida*,) to polyphagy (*O. crenata, O. minor, Ph. aegyptiaca, Ph. mutelii, Ph. ramosa*,). The typical feature of wild species is monophagy (*O. elatior, O. hederae, O. kochii*/**Figure 1B**/, *Ph. arenaria*/**Figure 1E**/) or narrow oligophagy (*O. alsatica*/**Figure 1C**/, *O. flava, O. alba, O. caryophyllacea, O. reticulata, Orobanche mayeri*/**Figure 1D**/) (**Figures 6** and **7**). In general, the hierarchical clustering of the genera *Orobanche* and *Phelipanche* reflects the known taxonomy especially in internal taxonomic grouping of genus (subsections). Distribution and composition of the minor clades was very stable in all cluster analyses performed. Two highly supported clades in the genus *Orobanche* within the wild group were discriminated (bottom half of **Figure 6**). The first clade (BP/AU 95/99) involves the species from three subsections, *Glandulosae* (*O. alba* and *O. reticulata*), *Orobanche* (*O. mayeri*), and *Galeatae* (*O. caryophyllacea*). The former two, however, did not cluster separately, since *O. mayeri* clustered closely with *O. reticulata*. The second clade (BP/AU 95/99) represents a monophyletic group and matches with the subsection *Orobanche*. In this clade, *O. kochii* is closer related to *O. flava* and more distant from *O. elatior*, which is closer to *O. alsatica*. Interestingly, *O. mayeri* (mentioned above) differed markedly from the morphologically difficult to assign *O. alsatica* aggregate, where it taxonomically belongs! Unexpectedly the yellow morph of *O. flava* clustered separately from the other color morphs of the well supported *O. flava* clade (**Figure 6**). Three well supported clades in the genus *Orobanche* were present in the weedy group (top half of **Figure 6**). One of these is the cluster of three aromatic species with differing taxonomy, *O. lutea* (*Galeatae*), *O. crenata* (*Speciosae*), and *O. ballotae* (*Minores*). Two further clades observed were the basal lineage corresponding to *O. foetida* (*Cruentae*) and the lineage with *O. minor* (*Minores*), and *O. cumana* and *O. cernua* (*Coerulescentes*). The separate clustering of *O. minor* was statistically sufficiently supported and was clearly separate from *O. cumana* and *O. cernua*. The latter two were also distinct from each other (**Figure 6**). Weedy species of the genus *Phelipanche* (section *Phelipanche*) clustered together well supported within the weedy cluster (**Figure 7**). Within this cluster, *Ph. mutelii* and *Ph. aegyptiaca* constitute one clade and *Ph. ramosa* relatively isolated another. The most distinct species *P. arenaria* (section *Arenariae*) clustered completely separate from the other *Phelipanche* spp. within the wild broomrape group (**Figures 4** and **7**), and constitutes a separate lineage either alone (**Figure 3**) or together with *O. hederae* (**Figure 4**).

### Synapomorphic Characters

Using Xcalibur software, we identified which VOCs are differentially emitted by species within the wild and the weedy clusters. Since these potential synapomorphic characters (shared, newly evolved features) could carry phylogenetic information, mapping the presence and absence of VOCs was realized in all 21 European broomrapes (including *Boulardia* and excluding *Cistanche*). Out of the 544 VOCs considered, two compounds, 2,3-butanediol and phytone (hexahydrofarnesyl acetone) were unique to the weedy species only. Species from the genera *Phelipanche* [except *Ph. arenaria* (!)] emitted both. 2,3-Butanediol occurred in the weedy group *O. cumana, O. cernua, O. minor, O. foetida, O. ballotae*, and non-broomrape *B. latisquama* and phytone occurred also in the weedy *O. minor, O. cumana*, and *O. cernua*. On the other hand, the sesquiterpene aristolene was exclusively emitted by *Orobanche* spp. with the exception *O. cumana* and *O. cernua*. 2-methylpropan-1-ol and the monoterpenoid eucalyptol were common VOCs in most *Orobanche* spp. and *Ph. arenaria* but they were absent in the weedy species.

## Discussion

### Phylogenetic Patterns within Broomrapes

A number of authors have reported on failed efforts to use floral scent chemistry for phylogenetic analysis (Williams and Whitten, 1999; Levin et al., 2003; Meekijjaroenroj et al., 2007) and as far as we know only two studies have yielded credible phylogenetic patterns (Barkman, 2001; Raguso et al., 2006). One possible explanation is that the number of species and especially the number of VOCs used in the studies that failed was quite low. The number of VOCs we have collected exceeds that of previous studies. The differences reflect probably unusually high scent diversity found in broomrapes and the high sensitivity due to thermal desorption. Our metabolomics approach enabled the use of the more complete VOC profile. Together this may have been decisive in generating sufficient data to allow for reliable statistical analysis and hence reliable phylogeny. The chemical and statistical analysis of the floral VOCs of 23 European broomrapes (including *Boulardia* and *Cistanche*) resulted in phylogenetic relationships that are offering new insights into broomrape phylogeny that can be tested with independent phylogenetic analyses. The key difference, in comparison with phylogenetic relations inferred from taxonomical (Beck-Mannagetta, 1930; Teryokhin, 1997) or recent molecular studies (Román et al., 2003; Manen et al., 2004; Schneeweiss et al., 2004; Schneeweiss, 2007; Park et al., 2008), is the separation of broomrapes into two main groups, a weedy (growing on annual and short lived perennial hosts) and a wild (growing on perennial hosts) group.

The clades now recognized are based on functional traits, such as host range and life history, but these clades are not easily diagnosed using morphological characters. It has been hypothesized that the host range and life history of broomrapes evolved in a correlated fashion (Schneeweiss, 2007). Our floral scent analyses underline that specialization for the type of host plant is indeed very likely the driving force of evolution within the broomrapes (**Figures 6** and **7**). However, also evolution of weediness seems to be an important driving force in speciation in the *Orobanche* and *Phelipanche* genera. Weedy broomrapes were placed into two independent phylogenetic clusters within the genus *Orobanche* (*O. cumana* group and *O. crenata group*), and into one within the genus *Phelipanche* (**Figures 6** and **7**). This suggests that weediness originated several times independently during the evolution of the broomrapes.

The level of host specialization (monophagy, oligophagy, polyphagy) seems to be less significant for the phylogenetic patterns than the host lifestyle (annual or perennial). For instance, in the group of weedy species of the genus *Orobanche*, host specialization ranges from monophagy (*O. cumana* – weedy forms on sunflower but wild forms on *Artemisia* spp.), through oligophagy (*O. cernua* – weedy forms on Solanaceae and wild forms on *Artemisia* spp. and some other Asteraceae) up to polyphagy (*O. minor* – various families) (Teryokhin, 1997). A similar situation occurs within the aromatic weedy species. *O. crenata* is a typical polyphagous species (Parker, 2009), *O. lutea* ranges from monophagy to oligophagy (Teryokhin et al., 1993; Piwowarczyk and Krajewski, 2014), while the non-weedy *O. ballotae* is strictly monophagous (Pujadas Salva, 1997) (**Figure 6**).

Support for life history of the host plant being a driving force in broomrape evolution and hence phylogeny is provided by species from the subsections *Orobanche, Galeatae*, and *Minores*. All species of the subsection *Orobanche* grow on perennial hosts, share one monophyletic clade with the exception *O. mayeri*, and all clusters just as expected based on the known taxonomical subsection structure (Teryokhin et al., 1993; Teryokhin, 1997). However, the other two subsections show clustering that is not consistent with existing taxonomy (**Figures 3, 4** and **6**). Such discordance has been also identified before by molecular-phylogenetic clades based on a broader species sampling (Manen et al., 2004; Schneeweiss et al., 2004). This is, for example, clear in the subsection *Minores* (Beck-Mannagetta, 1930) (**Figures 2** and **5**). Minores species *O. minor* groups with weedy species that grow on annual hosts, *O. ballotae* together with aromatic weeds on short-lived perennials, whereas *O. hederae* shares a clade with *Ph. arenaria* and both are growing on perennial hosts (**Figure 4**). Our data support Teryokhin et al. (1993) rather than Beck-Mannagetta (1930). For instance *O. hederae* placement in a separate subsection *Hederae* and not together with the rest of *Minores* is highly supported by our study. Phylogenetic hypotheses based on DNA sequence data also suggested the presence of heterogeneity in the *O. minor* clade (Schneeweiss, 2007). We propose that this unclear phylogeny can be resolved by using floral scents. Our data verified and supported also known morphological (Teryokhin et al., 1993) and molecular (Schneeweiss et al., 2004; Park et al., 2008; Castillejo et al., 2009) distinctions between the sections *Phelipanche* and *Arenariae*, and this were statistically supported here. Besides VOCs data placed interestingly *Phelipanche* within *Orobanche* (**Figures 3** and **4**).

**Figure 5** shows *B. latisquama* (syn. *Orobanche macrolepis*) as a phylogenetically clearly independent group, as was also concluded by Schneeweiss et al. (2004). On the other hand *B. latisquama* and *Ci. phelypea* are clearly part of the *O. cumana* lineage within the weedy broomrape group (**Figures 3** and **4**). The genus *Paulownia* has been placed in a clade with Orobanchaceae and Phrymaceae (Tank et al., 2006). This placement was confirmed also here (**Figures 2** and **3**). Surprisingly, *P. tomentosa*, a tree native to China, *M. cardinalis* (Phrymaceae) of North American origin, and *Antirrhinum* spp. (Veronicaceae) of Eurasian origin clustered together in the weedy *Phelipanche* spp. clade. This could point to a New World origin for *Phelipanche*, in contrast to the likely Old World *Orobanche* (Schneeweiss et al., 2004). The latter shared a mutual clade with the parasitic *Striga* spp. (**Figure 3**). *S. hermonthica* and *S. asiatica*, both with host preference for tropical cereal crops, grouped closely together, while *S. gesnerioides* parasitizing fabaceous host species clustered separately from the two cereal parasites.

The presence of aromatic VOCs can provide additional support for clades (Levin et al., 2003). Aromatic species were found in three different clusters, the *O. foetida, O. lutea*, and *O. caryophylacea* groups (**Figures 3, 4**, and **6**). The strong aromatic odor seems to be related to the typical habitat – open, sunny, and warm with abundant insect fauna – of these broomrapes. However, the very distinct scent of these aromatic broomrapes does not result in high insect diversity on the rewarding broomrape flowers (Tóth pers. obs.). Possibly, the strong fragrance acts as plant defense rather than pollinator lure as also demonstrated for aloe nectar (Johnson et al., 2006).

### Species Determination

In our study, the broomrapes are generally well differentiated by their floral scents and the samples of single species, even of individuals from different locations or years, clearly group together (**Figures 3, 4**, and **6**). As it is often unclear to what degree the host plant can influence parasite morphology (Musselman and Parker, 1982), and there is no cardinal feature that may be employed to distinguish species reliably, VOCs could be an alternative for solving taxonomical problems. For example, it is not easy to distinguish *O. elatior* (**Figure 1A**) from *O. alsatica* (**Figure 1C**) on the basis of morphological traits (Zázvorka, 2010) and the same is true for *O. alsatica* and *O. mayeri* (**Figure 1D**) (Piwowarczyk et al., 2014). Floral scents provide a useful tool to support species identification and clearly support the separation of these three into separate species (**Figures 3, 4**, and **6**). Interesting is the phylogenetic location of the rare *O. mayeri*. *O. mayeri* shares its habitat preference with *O. reticulata* with which it closely groups and has an aromatic scent which explains its phylogentic placement close to the aromatic group (*O. alba, O. caryophylacea*) (**Figure 6**). But its VOCs clearly separate *O. mayeri* from the morphologically similar *O. alsatica*. The most notable compounds are methyl 3-methylbutanoate (methyl isovalerate) and cuparene [1-methyl-4-(1,2,2-trimethylcyclopentyl)-benzene)]. Both are absent in *O. alsatica* but do occur in *O. mayeri*. Methyl isovalerate and cuparene are also present in the floral blend of *O. reticulata, O. alba* and *O. caryophylacea* and absent in *O. elatior* which shares a clade with *O. alsatica* (**Figure 6**). Morphological and evolutionary proximity of *O. mayeri* with *O. flava* (Zázvorka, 1997) is also rejected by our analysis. All of this suggests that taxonomy of *O. mayeri* could be revalued. Additional support for the reliability of our approach is represented by the explicit separation of *O. elatior* and *O. kochii* (**Figure 6**). The host of both species is *Centaurea scabiosa*. For a long time, these species were considered two color morphs (yellowish and carrot-red) of *O. elatior*, but recently the existence of two taxonomically distinct species was postulated (Zázvorka, 2010; Frajman et al., 2013), which is clearly supported by our VOC analysis. Another surprising, color related, separation was found in *O. flava*. The VOC profiles of yellow morphs differed from red wine-color and typical (ochreous-pink) morphs. The latter two created a well-supported distinct clade (**Figures 3, 4**, and **6**) and the distinct clustering of the yellow morph (including samples collected from different locations!) suggests the existence of a new subspecies. Majetic et al. (2007) found differences in floral VOCs of purple and white color morphs of *Hesperis matronalis* (Brassicaceae). They assumed that mutants (white morph) could divert compounds normally processed to form pigment to other pathways which could result in changes in type or amount of VOCs emitted. A similar mechanism may be responsible for the possible sub-species creation in *O. flava*. In addition to a number of minor quantitative differences in VOC amounts emitted by the two morphs, the typical morphs emit 6-methyl-5-hepten-2-ol while the yellow morph does not. This places the yellow morph of *O. flava* phylogeneticaly closer to *O. elatior*. *O. elatior* (**Figure 1A**) is yellowish and also missing 6-methyl-5-hepten-2-ol, whereas closely related carrot-red *O. kochii* (**Figure 1B**) emits this metabolite.

### Phylogenetic and Ecological Significance of VOC Differences

Floral VOCs are unique features and each broomrape species has its own blend, which results in the well supported separate clustering of the species. Two VOCs, 2,3-butanediol and phytone seem to represent a synapomorphic character as they are closely connected to weediness. Particularly 2,3-butanediol and its precursor acetoin seem to be important in this respect. Whereas acetoin is a common floral VOC, 2,3-butanediol is much less often reported (Knudsen et al., 2006). Indeed, while acetoin was emitted by all broomrapes, 2,3-butanediol occurred only in the weedy species. The reduction of acetoin to 2,3-butanediol (Ryu et al., 2003) seems to represent a novel evolutionary trait in these weedy species. Interestingly, both volatile are released abundantly by plant growth-promoting rhizobacteria (Rudrappa et al., 2010). Especially 2,3-butanediol is an essential component responsible for airborne chemical signaling between rhizobacteria and plants (Ryu et al., 2003). For instance, exogenous application of 2,3-butanediol to *Arabidopsis thaliana* seedlings promotes growth and induces systemic acquired resistance (Ping and Boland, 2004). The intriguing acquisition of 2,3-butanediol emission by the weedy broomrapes may be speculated to represent a strategy that results in improved growth of the broomrape host or the broomrape itself.

The sesquiterpene aristolene is a key feature of most *Orobanche* spp. Aristolene is known as floral VOC only from two families, Araceae and Nyctaginaceae (Knudsen et al., 2006). Root extracts of *Valeriana jatamansi* (Valerianaceae), containing 5.2% of this compound, were shown to have insecticidal activity (Dua et al., 2008). The aristolene in the flower VOC blend of the *Orobanche* spp. may have a similar function and protect the flowers against insect herbivory. The other compound only present in the weedy *Phelipanche* broomrapes is phytone. Phytone is a ubiquitous compound, occurring in plants and insects (Schulz et al., 2011). It is known for its allelopathic, antimicrobial, antifungal, and pheromonal activity (Cakir et al., 2005; Radulovic et al., 2006; Kalinová et al., 2009).

The presence of synapomorphic characters as well as the position in the HCD may help to predict potentially problematic future weedy broomrapes. *O. foetida* could serve as an example as this species became an agricultural problem just a few decades ago and before grew only on wild host plants (Rubiales et al., 2005). The floral VOCs in retrospect pre-determine *O. foetida* to be a weedy broomrape (**Figure 6**), which it indeed became. Looking to the future, *O. lutea*, a species with weedy volatile pattern (**Figure 6**), may be the next problematic species. Presently, it grows almost exclusively on the wild *Medicago falcata* (Piwowarczyk et al., 2011), but it could potentially become a serious weed of alfalfa and clover (Teryokhin, 1997). The clustering of *B. latisquama* and *Ci. phelypea* in the *O. cumana* lineage could also point to a risk that they may develop into future weeds.

The ultimate ecological relevance of flower VOCs is of course the mediation of the interaction between flowers and animal flower visitors (Raguso, 2008a). In Slovakia, the key broomrape pollinators are social wasps (*Dolichovespula norwegica, D. sylvestris, Vespula rufa* on *O. flava*, and *Polistes nimpha* on *O. alba, O. alsatica, O. lutea*), bumblebees (e.g., *Bombus lucorum, B. pascuorum, B. hortorum*, and *B. ruderalis* on *O. flava, O. lutea, O. alsatica, O. elatior, O. reticulata*), bees from the Halictidae and Colletidae (*O. alba, O. alsatica, O. elatior, O. flava, O. reticulata, Ph. ramosa*), and hoverflies (Syrphidae) (especially on *O. flava*). However, no social wasps and bumblebees were recorded on any weedy species. 2-methylpropan-1-ol together with acetic acid and 2-methylbutan-1-ol are highly attractive for wasps especially in a mixture (Landolt et al., 2007). While these three VOCs are common across the wild broomrapes (including *Ph. arenaria*!) as well as in *O. lutea* and *O. crenata* that locate in the “weedy” group, 2-methylpropan-1-ol is completely missing in the other weedy broomrapes. Eucalyptol, known as the main active ingredient of the bumblebee foraging alert pheromone (Granero et al., 2005), is present in the volatile blend of all broomrapes. Nevertheless, weedy species (e.g., *Ph. ramosa*) are not visited by bumblebees despite the fact that they are present and for example pollinate the crop (e.g., tomato) on which these broomrapes parasitise. Except β-myrcene, α-pinene, β-pinene, *p*-cymene, and limonene, which are present in all broomrapes, there are many other compounds that could be attractive for bees, e.g., geraniol, trans-linalool oxide, γ-terpinene and terpinyl acetate (Dobson, 2006; Dötterl and Vereecken, 2010) present in the volatile blend of wild species while they are absent in weedy species. The VOC blends of *Phelipanche* spp. contain markedly more benzenoids. Flowers of *Ph. ramosa* (**Figure 1F**) were only pollinated by sweet bees (Halictidae) and polyester bees (Colletidae). Theis (Theis, 2006) showed that sweet bees are attracted to phenylacetaldehyde, a very general insect attractant (El-Sayed et al., 2008). This VOC is very common also in *Phelipanche* spp. (except *Ph. arenaria*/**Figure 1E**/) but it is missing in wild broomrapes (except *O. alba*). In conclusion, the loss of pollination by social wasps and bumblebees in weedy broomrapes coincides with a change in floral VOCs. Moreover the majority of the weedy species (including *O. cumana, O. crenata, O. minor*) are blue or purple and these colors contribute minimally to pollinator attractiveness (Sun et al., 2005) while decreasing herbivore performance (Strauss and Whittall, 2006). On the other hand, blue and purple flowers have a higher fitness including higher rates of seed maturation in a short growing season (Whittall and Carlson, 2009) a feature vital for weedy species.

## Conclusion

We showed that floral VOCs can to distinguish broomrape species. The life history of the host plants seems to be the driving force in the evolution within these parasitic plants. In addition, the floral VOCs clearly separated the weedy broomrapes from the wild species. This and the synapomorphic characters associated with the weediness could be of help in forecasting potential future weed problems. The reproducible character of the VOC blends could be a useful tool to support the taxonomical phylogeny of the Orobanchaceae.

## Author Contributions

The authors have made the following declarations about their contributions: Conceived and designed the experiments: PT, HB. Performed the experiments: PT. Analyzed the data: PT, AU. Contributed reagents/materials/analysis tools: FV. Wrote the paper: PT, HB.

## Conflict of Interest Statement

The authors declare that the research was conducted in the absence of any commercial or financial relationships that could be construed as a potential conflict of interest.
