# Supplementary material for: Floral Volatiles in Parasitic Plants of the Orobanchaceae. Ecological and Taxonomic Implications
Source: Front Plant Sci. 2016 Mar 15;7:312. doi: 10.3389/fpls.2016.00312 (PMC4791402; doi:10.3389/fpls.2016.00312)
Supplement: Supplementary file 1 [file Table_1.PDF]

Supplementary Table 1 Plant species included in the floral VOC study with host plant species and geographical origin.

**Species/family**	**Host plant**	**Host family**	**Area of origin**	**Location co-ordinates /seeds origin**	**Altitudes (m a.s.l.)**	**Number of samples**
Parasitic plants - Orobanchaceae - Broomrapes - wild						
Boulardia latisquama F.W.Schultz	*Rosmarinus oficinalis*	Lamiaceae	Spain	37˚56'N04˚53'W	309	4
Cistanche phelypaea 1 (violacea) (Desf.) Hoffmanns. & Link	*Salsola* spp.	Chenopodiaceae	Spain	37˚01'N02˚26'W	281	1
Cistanche phelypaea 2 (lutea) (L.) Cout.	*Salsola* spp.	Chenopodiaceae	Spain	37˚01'N02˚26'W	281	2
Orobanche alba 1 Stephan&Willd.	*Thymus serpyllum*	Lamiaceae	Slovakia	48˚25'N 21˚46'E	160	2
O. alba 2 Stephan&Willd.	*Thymus serpyllum*	Lamiaceae	Slovakia	48˚28'N 20˚28'E	421	2
O. alsatica Kirschl.	*Puecedanum cervaria*	Apiaceae	Slovakia	47˚49'N 18˚38'E	193	2
O. ballotae A. Pujadas	*Ballota hirsuta*	Lamiaceae	Spain	37˚48'N05˚01'W	310	3
O. caryophyllacea 1 Sm.	*Gallium mollugo*	Rubiaceae	Slovakia	49˚12'N 18˚59'E	740	3
O. caryophyllacea 2 Sm.	*Gallium mollugo*	Rubiaceae	Slovakia	48˚36'N 20˚52'E	373	1
O. elatior Sutton	*Centaurea scabiosa*	Asteraceae	Slovakia	48°32'N18°32'E	740	4
O. flava 1 (typical) Mart.&F.W.Schultz	*Petasites albus*	Asteraceae	Slovakia	48˚58'N 19˚43'E	938	3
O. flava 1 (yellow) Mart.&F.W.Schultz	*Petasites albus*	Asteraceae	Slovakia	48˚58'N 19˚43'E	938	1
O. flava 2 (typical) Mart.&F.W.Schultz	*Petasites* spp.	Asteraceae	Slovakia	49˚00'N 19˚52'E	721	3
O. flava 2 (yellow) Mart.&F.W.Schultz	*Petasites* spp.	Asteraceae	Slovakia	49˚00'N 19˚52'E	721	1
O. flava 3 (typical) Mart.&F.W.Schultz	*Petasites albus*	Asteraceae	Slovakia	49˚15'N 19˚42'E	1001	3
O. flava 3 (red) Mart.&F.W.Schultz	*Petasites albus*	Asteraceae	Slovakia	49˚15'N 19˚42'E	1001	1
O. flava 4 (typical) Mart.&F.W.Schultz	*Petasites* spp.	Asteraceae	Slovakia	49˚12'N 18˚59'E	740	1
O. flava 4 (yellow) Mart.&F.W.Schultz	*Petasites* spp.	Asteraceae	Slovakia	49˚12'N 18˚59'E	740	2
O. hederae Duby	*Hedera helix*	Araliaceae	Netherlands	51˚57'N 05˚39'E	9	3
O. kochii F.W.Schultz	*Centaurea scabiosa*	Asteraceae	Slovakia	48˚27'N 20˚18'E	262	4
O. mayeri (Suess.&Ronninger) Bertsch	*Pimpinella major*	Apiaceae	Slovakia	48˚58'N 19˚42'E	1338	2
O. reticulata 1 Wallr.	*Carduus glaucinus*	Asteraceae	Slovakia	48˚58'N 19˚42'E	1338	1
O. reticulata 2 Wallr.	*Carduus glaucinus*	Asteraceae	Slovakia	48˚59'N 19˚36'E	1490	1
Phelipanche arenaria (Borkh.) Pomel	*Artemisia vulgaris*	Asteraceae	Slovakia	48˚41'N 18˚25'E	261	2
Parasitic plants - Orobanchaceae - Broomrapes - weedy						
O. cernua L.	*Lycopersicum esculentum*	Solanaceae	Netherlands (greenhouse)	Israel (2009) *Lycopersicum esculentum*		2
O. crenata Forssk.	*Vicia faba*	Fabaceae	Spain	37˚53'N04˚46'W	120	4

Supplementary Table 1 (continuation)

Species/family	Host plant	Host family	Area of origin	Location co-ordinates /seeds origin	Altitudes (m a.s.l.)	Number of samples
O. cumana Wallr.	*Helianthus annuus*	Asteraceae	Netherlands (greenhouse)	Bulgaria (2005)<br>*Helianthus annuus*		2
O. foetida Poir.	*Vicia faba*	Fabaceae	Netherlands (greenhouse)	Spain (2003)<br>*Vicia faba*		3
O. lutea 1 Baumg. (2009)	*Medicago falcata*	Fabaceae	Slovakia	48˚25'N 21˚46'E	160	2
O. lutea 2 Baumg. (2010)	*Medicago falcata*	Fabaceae	Slovakia	48˚25'N 21˚46'E	160	3
O. minor Sm.	*Trifolium repens*	Fabaceae	Netherlands (greenhouse)	England (2009)<br>*Trifolium pratense*		4
P. aegyptiaca (Pers.) Pomel	*Lycopersicum esculentum*	Solanaceae	Netherlands (greenhouse)	Israel (2003)<br>*Lycopersicum esculentum*		6
P. mutelii (F.W.Schultz) Pomel	*Brassica oleracea*	Brassicaceae	Netherlands (greenhouse)	Israel (2000)<br>*Lycopersicum esculentum*		3
P. ramosa (L.) Pomel	*Lycopersicum esculentum*	Solanaceae	Netherlands (greenhouse)	Slovakia (2007)<br>*Nicotiana tabaccum*		5
Parasitic plants - Orobanchaceae - Witchweeds						
Striga asiatica (L.) Kuntze	*Oryza sativa*	Poaceae	Netherlands (greenhouse)	Tanzania (2007)<br>*Zea mays*		3
S. gesnerioides (Willd.) Vatke	*Vigna unguiculata*	Fabaceae	Netherlands (greenhouse)	Nigeria (2005)<br>*Vigna unguiculata*		1
S. hermonthica (Delile) Benth.	*Oryza sativa*	Poaceae	Netherlands (greenhouse)	Mali (2005)<br>*Sorghum bicolor*		2
Parasitic plants - Cynomoriaceae						
Cynomorium coccineum L.	*Arthrocnemum glaucum*	Chenopodiaceae	Spain	37˚01'N02˚26'W	281	5
Non parasitic plants - Phrymaceae						
Mimulus cardinalis Dougl.&Benth.	-	-	Netherlands (greenhouse)	Laura Ashley,<br>UK (2009)		3
Mimulus luteus (guttatus) tigrinus DC.	-	-	Netherlands (greenhouse)	Buzzy seeds,<br>The Netherlands (2009)		4
Non parasitic plants - Veronicaceae						

Supplementary Table 1 (continuation)

Species/family	Host plant	Host family	Area of origin	Location co-ordinates /seeds origin	Altitudes (m a.s.l.)	Number of samples
Antirrhinum majus nanum L.	-	-	Netherlands (greenhouse)	Buzzy seeds, The Netherlands (2009)		4
Antirrhinum majus pumilum L.	-	-	Netherlands (greenhouse)	Buzzy seeds, The Netherlands (2009)		2
Non parasitic plants - Paulowniaceae						
Paulownia tomentosa (Thunb.) Steud.	-	-	Netherlands	51˚57'N 05˚39'E	9	4
