# Supplementary material for: Floral Volatiles in Parasitic Plants of the Orobanchaceae. Ecological and Taxonomic Implications
Source: Front Plant Sci. 2016 Mar 15;7:312. doi: 10.3389/fpls.2016.00312 (PMC4791402; doi:10.3389/fpls.2016.00312)
Supplement: Supplementary file 2 [file Table_2.PDF]

Supplementary Table 2

List of VOCs emitted by selected *Orobanche* spp. and *Phelipanche* spp. and two outgroup species. Data are based on floral metabolites emitted by *O. alba* (OA), *O. flava* (OF), *O. elatior* (OE), *O. reticulata* (OR), *Ph. ramosa* (PR), *Ph. aegyptiaca* (PAE), *Mimulus luteus* (ML), and *Paulownia tomentosa* (PT). VOCs are listed according to calculated retention indexes (RI); black dot means presence of particular VOC.

RI[a]	Volatile organic compound[b, c]	Occurrence							
		OA	OF	OE	OR	PR	PAE	ML	PT
461	Ethyl alcohol*	●	●	●	●	●	●		●
487	Propan-2-one*	●	●	●	●	●	●		●
583	Acetic acid *	●	●	●	●			●	●
589	Vinyl ethanoate							●	
590	tert-Butylmethylether					●			
600	Hexane*							●	
602	3-Methylpentane					●	●		
607	1-Hexene					●			
634	2-Methylpropan-2-ol					●			
654	2-Methylpropanal				●				
657	(E) 2-Butenal				●				
660	(Z) 2-Butenal				●				
666	Butan-2-one*			●	●	●			
671	2-methyl-3-buten-2-ol			●					
672	Ethyl acetate*	●	●	●	●	●	●	●	
674	Ethylcyclobutane					●			
674	2-Ethoxy-2-methyl-propane				●				
675	2-Methylpropan-1-ol (isobutanol)*	●	●	●	●				
677	Methylcyclopentane				●	●	●		
680	3-Methoxy-1-pentene					●			
682	2-Buten-1-ol				●				
685	3-Methylbutan-2-one					●			
686	3-Methylbutanal *	●	●	●	●	●	●		●
688	Benzene					●	●	●	
688	Butan-1-ol *	●	●	●	●	●			
689	2-Methylbutanal	●	●	●	●				●
690	3-Methylhexane					●	●		
690	(E)-2-Methyl-2-butenal		●						
690	3,4-Dihydropyran	●		●					
691	2,3-Dihydro-5-methylfuran		●						●
692	1-Methoxypropan-2-ol*		●	●	●		●	●	●
692	1-Methoxy-2-methylbutane	●							
694	1-Penten-3-ol*	●	●	●	●	●	●		
696	Methyl 2-methylpropionate				●				
696	Pentan-2-one	●	●	●		●			
696	Pentan-3-one								●
698	1-Hepten	●	●	●	●	●			
697	Isopropylcyclobutane				●				

Supplementary Table 2 (continuation)

RI[a]	Volatile organic compound[b, c]	Occurrence							
		OA	OF	OE	OR	PR	PAE	ML	PT
699	Pentanal*	●	●	●	●	●	●		
700	Heptane*	●	●	●	●	●	●	●	●
701	2-methyl-1,4-hexadiene		●						
708	3-Hydroxybutan-2-one (acetoin)*	●	●	●	●	●	●	●	
713	Methyl methacrylate	●		●		●			
715	Propyl acetate					●			
718	2,4-Dimethylfuran*	●	●	●	●	●	●		
721	Methyl butanoate							●	●
725	2-Methylbutanenitrile		●	●	●				
727	Cyclohexylmethane			●					
729	Methylcyclohexane			●		●	●		
731	1,1-Diethoxyethane		●		●				
732	2,5-Dimethylhexane					●			
733	3-Methylpentan-2-one			●					
734	3-Methylbutan-1-ol*	●	●	●	●	●	●		●
735	Propylene glycol								●
739	2-Methylbutan-1-ol*	●	●	●	●	●	●	●	●
742	4-Methylpentan-2-ol*	●	●	●	●	●	●		
744	Pyridine					●			
746	2-Methyl-2-butenal*	●	●	●	●	●	●		●
748	2,3,4-Trimethyl-pentane					●	●		
753	3-Methylpentan-2-one			●					
754	2-Methylpropanoic acid				●				
755	3,3-Dimethylhexane					●	●		
757	3-Methyl-1-heptene			●					
757	(E)-2-Pentenal	●							
759	2,3-Dimethylhexane					●	●		
760	4-Methylheptene*	●		●					
765	2-Methylheptane						●	●	
767	Pentan-1-ol*	●	●	●	●	●	●		
770	4-Methylheptane	●		●		●	●		
774	Toluene*	●	●	●	●	●	●	●	●
776	2-Methyl-2-buten-1-ol			●			●		
777	2,3-butanediol*					●	●	●	
778	Methyl 3-methylbutanoate*	●	●		●				
780	Methyl 2-methylbutanoate						●	●	●
784	Pentane-2,4-dione*	●	●	●	●	●	●	●	
785	Ethylhexanol*								●
787	3-Methyl-2-butenal*	●	●	●	●				
789	Hexan-2-one					●			
789	2-Methyl-1-heptene	●		●					
791	Hexan-2-one	●	●	●					
793	1-Octene*	●	●	●	●	●	●		
793	Ethyl butanoate							●	

Supplementary Table 2 (continuation)

RI[a]	Volatile organic compound[b, c]	Occurrence							
		OA	OF	OE	OR	PR	PAE	ML	PT
794	Cyclopentanone			●					
797	(Z)-3-Hexenal						●		
798	Hexanal*	●	●	●	●	●	●		●
800	Octane*				●		●	●	
805	Butyl acetate							●	
807	2-Methyldihydro-3(H)-furanone					●			
813	Butyl acetate	●	●	●		●	●		
813	Methyl pentanoate								●
817	2-Propyltetrahydrofuran					●			
824	2,4-Dimethylheptane*	●	●	●	●	●	●		
828	2-Methyl-4-propyloxetane			●					
833	3-Methylbutanoic acid	●	●			●	●		
837	4-Methylpentan-1-ol			●					●
838	Furan-2-carbaldehyde					●	●		
839	4-Hydroxy-4-methylpentan-2-one						●		
841	Ethylcyclohexane					●			
842	2,4-Dimethyl-1-heptene					●			
844	2-Methylbutanoic acid				●		●	●	
845	2,4-Dimethylheptene	●	●	●					
849	3-Methylbutyl acetate		●						
851	(Z)-3-Hexen-1-ol*					●	●		
853	Pentanedial								●
854	2-Methylbutanal oxime		●						
854	(E)-2-Hexenal					●	●		●
857	Chlorobenzene*	●	●	●					
858	(E)-3-Hexen-1-ol*				●	●	●	●	
859	(Z)-3-Hexenal					●			
860	cis-2,3-Epoxyoctane		●	●					
860	2,3-Dimethylheptane	●							
863	2-Methylhexan-3-ol				●				
864	Methyl 2-methylene butanoate						●		●
866	4-Methyloctane	●	●	●	●				
867	Hexan-1-ol*		●	●	●	●	●		
869	Pentanoic acid						●		
870	1-Ethylbenzene*	●	●	●	●	●	●	●	
872	3-Ethylpentan-2-one					●			
874	3-Methyloctane			●					
877	1,4-Dimethylbenzene*	●	●	●	●	●	●	●	●
879	4-Methylhexanal						●		
881	2-Hepten-4-ol				●				
884	2,4-Hexanedione			●	●				
886	1-Hepten-3-ol					●			
888	Methoxyphenyl oxime					●	●	●	
888	Heptan-3-one*	●	●	●	●				

Supplementary Table 2 (continuation)

RI[a]	Volatile organic compound[b, c]	Occurrence							
		OA	OF	OE	OR	PR	PAE	ML	PT
890	4-Isopropylcyclohexanol				●				
891	Heptan-2-one		●	●			●		
892	1-Nonene*	●			●	●	●		●
895	Methyl 2-hydroxy-3-methylbutanoate	●							
895	1-Nonyne		●						
896	5-Hepten-2-one						●		
900	Nonane*				●			●	
900	Ethenylbenzene*	●	●	●	●	●	●	●	●
903	1,2-Dimethylbenzene*			●		●	●	●	
903	Heptanal*	●	●		●	●	●		●
904	(E)-2-methylcyclopentanol acetate					●			
907	Butyl propionate						●		
907	2-Butoxyethanol	●	●	●		●		●	
910	(E)-2-methyl-2-butenoic acid						●		
912	2-Methylpropyl 2-methylpropanoate				●				
912	(E,E)-2,4-Hexadienal					●	●		
917	Dimethyl sulfone						●	●	
919	Dihydrofuran-2(3H)-one				●	●			
923	Methyl hexanoate						●	●	●
929	2,5-Hexanedione	●	●		●				
931	α-Thujene						●		
934	Cumene			●		●	●	●	
935	2,6-Dimethyloctane		●						
936	β-Thujene	●		●					
941	4-Methyl-2-heptanone	●		●	●				
943	1-Butoxy-2-propanol		●						
947	α-Pinene*	●	●	●	●	●	●	●	●
954	Isobutyl butyrate		●						
955	2,3,3-Trimethyl-2-pentanol						●		
956	6-Methylheptan-2-one						●		
956	2,4-Thujadiene	●							
957	2-Ethylhexanal						●		
957	4-Ethyloctane		●						
958	5-Ethylfuran-2-(5H)-one						●		
959	(E)-2-Heptenal					●			
962	1-Propylbenzene*			●	●	●	●		
962	(Z)-2-Heptenal	●			●				
964	4-Methylnonane		●						
966	5-ethyl-2-furanone							●	
969	Heptan-1-ol		●			●			
969	Hexanoic acid	●					●	●	
972	m-Ethylmethylbenzene		●	●	●				
973	Benzaldehyde*	●	●	●	●	●	●	●	●
978	Pseudocumene		●	●		●	●	●	

Supplementary Table 2 (continuation)

RI[a]	Volatile organic compound[b, c]	Occurrence							
		OA	OF	OE	OR	PR	PAE	ML	PT
979	Phenol						●		
979	1-Octen-3-ol*	●	●	●	●				●
983	1-Ethyl 2-methylbenzene						●		
986	3-octanone								●
987	6-Methyl-5-hepten-2-one*	●	●		●	●	●		
992	β-Myrcene*	●	●	●	●	●	●		●
995	Methoxymethylbenzene								●
996	2,4,6-Trimethylpyridine				●		●		
995	3-Isobutyl-cyclohexene				●				
996	Ethyl hexanoate							●	
997	2,4,6-Trimethylpyridine				●		●		
998	2,2,4,6,6-Pentamethylheptane			●		●			
1000	Decane*	●	●	●	●	●	●	●	
1005	1,3,5-Trimethylbenzene			●		●	●	●	
1005	Octanal*	●	●	●	●	●	●	●	●
1008	2-Carene						●		
1011	2,6-dimethyloctane	●							
1011	1-(2,2-Dimethylcyclopentyl) ethanone		●						
1011	2,2,4-Trimethylpentylvinylether			●					
1018	α-Terpinene					●	●		
1021	3-Carene		●		●	●			
1028	1,4-Dichlorobenzene			●					
1029	2-Ethylhexan-1-ol*	●	●	●	●	●	●	●	●
1037	p-Cymene*	●	●	●	●	●	●	●	
1037	1,2,3-Trimethylbenzene*	●	●	●	●	●	●	●	
1042	Limonene*	●	●	●	●	●	●	●	
1043	Methyl 2-ethylhexanoate		●		●				
1045	Phenylmethanol					●	●		●
1045	β-Phellandrene*	●				●	●		
1046	2,4,6-Trimethyl-1,6-heptatrien-4-ol						●		
1047	Eucalyptol*	●	●	●	●	●	●		
1049	2,3-Dihydro-1H-indene						●		
1050	2,3-Dimethylcyclohexanol			●					
1052	1-Octyn-3-ol				●				
1052	E,E-Alloocimene			●					
1052	trans-β-Ocimene	●							●
1054	3-Methylbutyl butyrate		●						
1055	5 Caranol				●				
1056	2-Phenylacetaldehyde*	●				●	●	●	
1057	1-Methyl-3-propylbenzene						●		
1060	Ocimene*				●	●			
1061	3,6-Dimethyldecane	●	●						
1063	1,4-Diethylbenzene						●		
1064	1-Pentyl-1H-pyrrole				●				

Supplementary Table 2 (continuation)

RI[a]	Volatile organic compound[b, c]	Occurrence							
		OA	OF	OE	OR	PR	PAE	ML	PT
1067	2-Methyldecane	●	●	●					
1071	γ-Terpinene	●		●					
1073	2(E)-2-Nonen-1-ol				●				
1075	Dihydromyrcenol	●	●						
1075	Octan-1-ol				●	●			
1080	Ethanone, 2-(formyloxy)-1-phenyl-						●		
1080	1-Phenylethanone*	●	●			●	●	●	●
1082	cis-Linalool oxide	●							●
1087	4-Ethyl-o-xylene						●		
1097	2,6-Dimethyl-1,5,7-octatrien-3-ol					●			
1100	trans-Linalool oxide	●	●	●					
1100	2-Propylheptanol				●				
1100	Undecane*				●		●		
1103	β-Linalool		●						
1107	Methyl benzoate					●	●	●	●
1108	Nonanal*	●	●	●	●	●	●		
1114	cis-β-Terpineol	●							
1116	Trimethyl phosphate					●	●		
1117	2-Butyl-2-hexanol						●		
1120	4-Acetyl-1-methylcyclohexene				●				
1122	4-Isopropenylcyclohexanone	●		●					
1124	Methyl octanoate							●	
1126	7H-Purin-6-ol				●				
1127	E,E-Cosmene						●		
1130	2-Phenylethanol	●				●	●		
1134	trans-Pinocarveol	●				●	●		
1135	1,2,4,5-Tetramethylbenzene		●				●		
1145	cis-Limonene oxide		●						
1151	Methyl nicotinate								●
1155	Lilac aldehyde A	●							
1156	Oxoisophorone*	●				●	●		
1161	2-Isopropyl-3-methylcyclohexanol				●				
1163	(Z)-3-Nonen-1-ol			●					
1165	Lilac aldehyde C	●							
1166	1-ethenyl-4-methoxy-benzene								●
1166	(E)-2-Nonenal					●			
1167	Benzyl acetate						●		
1171	R-camphor*	●	●	●	●				
1172	Nonan-1-ol					●			
1173	2-Tridecen-1-ol					●			
1174	Methyl 2-((1R,2R)-2-hexylcyclopropyl)-acetate					●			
1175	2-Decen-1-ol*	●	●	●	●	●	●		
1178	1,4-dimetoxy-benzene								●
1178	Sabina ketone		●						

Supplementary Table 2 (continuation)

RI[a]	Volatile organic compound[b, c]	Occurrence							
		OA	OF	OE	OR	PR	PAE	ML	PT
1180	3,5,5-Trimethyl-1,4-cyclohexanedione	●				●	●		
1183	Ethyl benzoate							●	
1186	Epoxylinalool	●							
1187	3,6-Dimethyl-2-octanone						●		
1190	Menthol	●	●	●	●	●			
1190	3-Methylbutyl (Z)-2-methyl-2-butenoate						●		
1193	1-Dodecene	●							
1194	Ethyl octanoate							●	
1196	trans-3-Caren-2-ol					●			
1200	Dodecane*	●	●	●		●	●		
1206	Dill ether					●			
1208	Decanal*	●	●	●	●	●	●	●	●
1213	Methyl salicylate								●
1214	Naphthalene		●	●			●		
1215	(E)-2-Octenyl 2-methyl-(E)-2-butenoate						●		
1223	Itaconic acid diethylester*	●	●		●	●	●		
1232	4-tert-Butylcyclohexanol		●						
1233	N-Phenyl formamide					●	●		
1235	Citronellol			●					
1237	3-Methyl-2-butenoic acid, 3-methylbut-2-enyl ester						●		
1238	Fenchyl acetate		●						
1238	(Z)-3-Hexenyl 3-methylbutanoate				●				
1245	Borneol				●				
1250	Benzothiazole					●	●		
1255	Geraniol*	●	●						
1256	Diethyl ethylidenemalonate					●	●		
1261	Benzyl propionate						●		
1262	Carvone		●						
1268	4-methoxy-2-methyl-benzenamine								●
1270	Nonanoic acid	●							
1271	2-Butyloctan-1-ol		●						
1273	Decan-1-ol	●							
1275	(Z)-5-Tridecene					●			
1281	β-Homocyclocitral								●
1282	2,3,5,8-Tetramethyldecane			●			●		
1283	(Z)-4-Tridecene					●			
1285	5-Butyl-4-nonene					●	●		
1287	3,7-Dimethyl-6-octen-1-ol				●				
1295	Isopulegol acetate		●						
1296	4-Ethylguaiacol	●							
1300	Tridecane*	●	●	●	●	●	●	●	
1301	Estragole								●
1304	Benzyl 2-methylpropenoate						●		
1307	Limonene dioxide		●						

Supplementary Table 2 (continuation)

RI[a]	Volatile organic compound[b, c]	Occurrence							
		OA	OF	OE	OR	PR	PAE	ML	PT
1311	Isobornyl acetate		●						
1324	Methyl decanoate							●	
1326	1-Methylnaphthalene						●		
1330	(E)-9-oxo-2-Decenoic acid					●			
1339	Benzyl 2-methylpropenoate						●		
1336	4-vinylguaiacol*	●							
1342	2,2,4,4,6,8,8-Heptamethylnonane		●						
1346	Methyl 2-methoxybenzoate								●
1353	4-tert-Butylcyclohexyl acetate		●						
1351	Benzyl butyrate						●		
1352	7-Methyl-Z-tetradecen-1-ol acetate					●			
1353	Benzyl glycolate						●		
1353	Cyclohexanone, 2(2-butynyl)	●							
1364	Delta-elemene	●							
1368	Terpinyl acetate	●	●	●	●				
1372	2,6,8-Trimethyldecane		●						
1374	β-Maaliene				●				
1380	Farnesan		●						
1390	o-tert-Butylcyclohexyl acetate		●						
1392	3-Hydroxy-2,2,4-trimethylpentyl ester of isobutanoic acic					●	●		
1393	Methyl 4-methoxybenzoate								●
1394	Ethyl decanoate							●	
1399	Methyl cinnamate						●		●
1400	Tetradecane*	●	●	●	●	●			
1404	Benzyl pentanoate						●		
1412	Di-epi-alpha-cedrene	●		●	●				●
1416	Dodecanal*		●		●	●	●		
1418	α-Panasinsen	●			●				
1419	2,4,7,9-Tetramethyl-5-decyn-4,7-diol					●			
1422	2-Methyl-1-dodecanol		●						
1422	β-Elemene	●							
1427	cis-Thujopsene	●							
1432	Vanillin	●							
1435	1-Phenoxybenzene		●						
1438	β-Gurjunene				●				
1439	β-Neoclovene	●							
1439	Aromadendrene			●					
1441	2-Methyl-Z-4-tetradecene		●						
1449	Cyclobutanecarboxylic acid, benzyl ester						●		
1452	8-Isopropyl-1-methy-[d]…….	●			●				
1453	Longifolene		●	●					
1458	α-Cedrene			●					●
1458	trans-Geranyl acetone*		●			●	●		
1461	Thujopsene	●			●				●

Supplementary Table 2 (continuation)

RI[a]	Volatile organic compound[b, c]	Occurrence							
		OA	OF	OE	OR	PR	PAE	ML	PT
1463	β-Farnesene								●
1464	β-Caryophyllene	●							
1467	Geranyl acetone				●				
1469	β-Cedrene		●						●
1471	Alloaromadendrene				●				
1478	Viridiflorene	●					●		
1479	Aristolene*	●	●	●	●				
1479	Dodecan-1-ol		●			●			
1484	Calamenene	●							
1487	2,6-di-t-butyl-p-benzoquinone		●		●	●	●		
1489	β-Guaiene	●			●				
1490	1-Pentadecene		●						
1500	Pentadecane*	●	●	●	●	●	●	●	
1508	S,Z-α-Bisabolene					●	●		
1509	α-Cetone		●						
1509	β-Ionone*								●
1510	Benzyl (E)-2-methyl-2-butenoate						●		
1511	γ-Himachalene	●			●				
1515	Methyl dodecanoate						●		
1516	Pentadecanal					●			
1516	γ-Cadinene	●	●		●				●
1519	Eremophilene					●	●		
1523	δ-Cadinene	●		●	●				
1523	Methyl dodecanoate							●	
1525	Methyl 10-hydroxydecanoate					●			
1526	β-Bisabolene								●
1540	Epiglobulol	●			●				
1544	Selina-3,7 (11)-diene	●							
1542	2,6,10-Trimethyltetradecane		●			●	●		
1551	Cuparene	●			●				
1553	β-sesquiphellandrene								●
1554	Lilial		●						
1558	α-Selinene	●							
1561	Globulol				●				
1576	β-Himachalene	●							
1592	2-Hexyl-1-octanol		●						
1598	8-Hydroxy-3-methylisochroman-1-one	●							
1600	Hexadecane*	●	●	●	●	●	●		●
1604	2,2,4-trimethyl-3-carboxyisopropylpentanoic acid isobutyl es						●		●
1609	Ethyl dodecanoate							●	
1615	5-Methoxy-6,7-dimethylbenzofuran	●							
1615	α-Cedrene epoxide		●						
1617	2-Phenylethyl (E)-2-methyl-2-butenoate						●		
1619	Geranyl isovalerate					●			

Supplementary Table 2 (continuation)

RI[a]	Volatile organic compound[b, c]	Occurrence							
		OA	OF	OE	OR	PR	PAE	ML	PT
1627	Isopropyl dodecanoate		●	●	●				
1630	Cyclopropane-1,2,3-tricarboxylic acid, triethyl ester					●			
1649	2,6,10-Trimethylpentadecane		●	●					
1659	6-Isopropenyl-4,8a-dimethyldecahydro-1-naphthalenol						●		
1668	Ethyl citrate					●			
1669	α-Cadinol	●							
1681	1-Heptadecene					●			
1681	Clavatol	●	●		●				
1700	Heptadecane*	●	●	●	●	●	●	●	
1706	2,6,10,14-Tetramethylpentadecane		●	●					
1713	(7a-Isopropenyl-4,5-[e]…….		●						
1719	Methyl tetradecanoate						●	●	●
1721	4,8a-Dimethyl-6-[f]…….		●		●				
1725	2-Methyl-4-(2,6,6-trimethylcyclohex-1-enyl)but-2-en				●				
1745	Aristolone	●							
1747	1-Isopropyl-4,8-dimethylspiro[4,5]dec-8-en-		●						
1789	α-Hexylcinnamaldehyde		●						
1794	Ethyl tetradecanoate							●	
1799	1-Phenyl-1,3,3-trimethylindane					●			
1800	Octadecane*	●	●	●	●	●	●	●	●
1810	Benzyl benzoate						●		
1810	2,6,11,15-Tetramethylhexadecane					●			
1813	Phytane		●						
1815	tert-Hexadecanethiol				●				
1822	Isopropyl tetradecanoate*	●	●	●	●	●			●
1839	Guaiazulene	●							
1852	Hexahydrofarnesyl acetone					●	●		
1888	Butyl tetradecyl phthalate								●
1900	Nonadecane*					●		●	●
1900	Dibutyl phthalate*	●	●	●	●	●			
1994	Ethyl hexadecanoate							●	
2000	Eicosane*	●	●	●	●	●	●	●	●
2029	Isopropyl hexadecanoate*	●	●	●	●	●			●
2100	Heneicosane*	●	●	●	●	●	●	●	●
2125	10-Methyl heneicosane	●							
2200	Docosane*	●	●	●	●	●		●	●
2244	3-Ethyl-3-hydroxy-androstan-17-one					●			
2300	Tricosane*	●	●	●	●	●	●	●	
2400	Tetracosane*	●	●	●	●	●	●		

Notes

[a] VOCs are listed according to calculated Kovats retention indices (RI). Calculation was done for capillary column RTX-5MS based on retention times of alkanes

Notes (continuation)

[b] Asterisk***** indicate VOCs which were checked by authentic standards and agreement with mass spectral libraries

[c] The most of the compounds were annotated by comparing the mass spectra with mass spectral libraries (Wiley 7th edition and NIST08), and by comparing calculated retention indices with those given by NIST08, Adams (2007) and El-Sayed (2012). The annotation of many compounds was verified using an in house developed mass spectra/RI library at Wageningen UR.

[d] 8-Isopropyl-1-methyl-1,2,3,4-tetrahydronaphthalen

[e] (7a-Isopropenyl-4,5-dimethyloctahydroinden-4-yl) methanol

[f] 4,8a-Dimethyl-6-(2-methyl-oxiram-2yl)-4a,5,6,7,8,8a-hexahydro-1H-naphthalen-2-one
