# Supplementary material for: Floral Volatiles in Parasitic Plants of the Orobanchaceae. Ecological and Taxonomic Implications
Source: Front Plant Sci. 2016 Mar 15;7:312. doi: 10.3389/fpls.2016.00312 (PMC4791402; doi:10.3389/fpls.2016.00312)
Supplement: Supplementary file 3 [file Table_3.PDF]

Supplementary Table 3.

Mutual VOCs emitted by all broomrapes (*Orobanche* spp. and *Phelipanche* spp.)

(data are based on VOCs emitted by 20 different broomrapes studied)

RT[a]	RI[a]	VOC[b]	Formula	Functional group
2,38	461	Ethyl alcohol	C2H6O	Alcohols
2,75	487	Acetone	C3H6O	Ketones
4,33	663	Acetic acid	C2H4O2	Carboxylic acid
4,88	672	Ethyl acetate	C4H8O2	Esters
5,83	686	3-methylbutanal	C5H10O	Aldehydes
6,80	699	Pentanal	C5H10O	Aldehydes
6,84	700	Heptane	C7H16	Hydrocarbons-Alkanes
7,05	711	Acetoin	C4H8O2	Diverse functional groups
7,62	735	3-methylbutanol	C5H12O	Alcohols
7,71	739	2-methylbutan-1-ol	C5H12O	Alcohols
7,75	742	4-methylpentan-2-one	C6H14O	Ketones
8,35	767	Pentan-1-ol	C5H12O	Alcohols
8,46	774	Toluene	C7H8	Aromatic hydrocarbons
8,70	784	Pentane-2,4-dione	C5H8O2	Ketones
8,89	793	1-Octene	C8H16	Hydrocarbon-Alkenes
9,09	800	Hexanal	C6H12O	Aldehydes
10,52	870	1-Ethylbenzene	C8H10	Aromatic hydrocarbons
10,68	878	1,4-Dimethylbenzene	C8H10	Aromatic hydrocarbons
11,14	900	Ethenylbenzene	C8H8	Aromatic hydrocarbons
12,01	947	1R-alpha-pinene	C10H16	Hydrocarbons-Monoterpenes
12,53	973	Benzaldehyde	C7H6O	Aldehydes-Monoterpene
12,80	989	Sulcatone	C8H14O	Ketones
13,02	1000	Decane	C10H22	Hydrocarbons-Alkanes
13,12	1005	Octanal	C8H16O	Aldehydes
13,54	1029	2-Ethylhexan-1-ol	C8H18O	Alcohols
13,74	1042	Limonene	C10H16	Hydrocarbons-Cyclic Terpenes
14,90	1108	Nonanal	C9H18O	Aldehydes
15,97	1175	2-Decen-1-ol	C10H20O	Alcohols
16,53	1210	Decanal	C10H20O	Aldehydes
17,88	1300	Tridecane	C13H28	Hydrocarbons-Alkanes
20,62	1500	Pentadecane	C15H32	Hydrocarbons-Alkanes
21,85	1600	Hexadecane	C16H34	Hydrocarbons-Alkanes
23,04	1700	Heptadecane	C17H36	Hydrocarbons-Alkanes
24,16	1800	Octadecane	C18H38	Hydrocarbons-Alkanes
25,23	1900	Dibutyl phthalate	C16H22O4	Esters
26,30	2000	Eicosane	C20H42	Hydrocarbons-Alkanes
27,26	2100	Heneicosane	C21H44	Hydrocarbons-Alkanes
28,28	2200	Docosane	C22H46	Hydrocarbons-Alkanes
29,48	2300	Tricosane	C23H48	Hydrocarbons-Alkanes
30,60	2400	Tetracosane	C24H50	Hydrocarbons-Alkanes

[a] VOCs are listed according to retention time (RT) and retention indices (RI)

[b] VOCs were checked by authentic standards, known RI and by agreement with mass spectral libraries
